# Dietary Modulation of the Gut Microbiota in Dogs and Cats and Its Role in Disease Management

**DOI:** 10.3390/microorganisms13122669

**Published:** 2025-11-24

**Authors:** Benlu Yang, Shengwei Zhong, Jue Wang, Wanting Yu

**Affiliations:** College of Animal Science and Technology, Jiangxi Agricultural University, Nanchang 330045, China; yangbenlu2003@outlook.com (B.Y.); zhongshengwei2021@163.com (S.Z.)

**Keywords:** gut microbiota, nutritional therapy, disease, fecal microbiota transplantation, dog, cat

## Abstract

Food has a massive influence on the gut microbiota and is one of the most useful therapeutic levers in disease. Recent developments have highlighted how macronutrient balance, food format, and functional ingredients can regulate microbial diversity, metabolism, and host physiology in companion animals such as dogs and cats. This narrative review condenses evidence on the bidirectional gut microbiota–diet connection and on nutritional therapy for gastrointestinal, metabolic, renal, hepatic, and immune-mediated disorders. Protein-based diets including high or hydrolyzed protein, omega-3 acids, fermentative fiber, and probiotics can positively affect microbial composition, stimulate short-chain fatty acid synthesis, and enhance intestinal barrier functions. Conversely, excess fats or refined carbohydrates may cause dysbiosis, inflammation, and metabolic imbalances. Numerous studies have shown that therapeutic nutrition—e.g., low-protein renoprotective, hepatoprotective antioxidants, and allergen-elimination diets—holds enormous potential for treatment. In addition, fecal microbiota transplantation (FMT) can be used as an additive therapy for resistant gastrointestinal illnesses. Despite these developments, constraints remain in terms of standardization, study duration, and species-specific data, especially for cats. This review underscores dietary modification as a clinically actionable tool for microbiota-targeted therapy and calls for integrative, multi-omics research to translate microbiome modulation into precision nutrition for companion animals.

## 1. Introduction

The gut in dogs and cats hosts a dynamic, multifaceted microbial ecosystem that is crucial for digestive function, immune modulation, metabolic control, and defense. As in humans, perturbations in the gut microbiome of companion animals have been linked to various chronic diseases, including inflammatory bowel disease (IBD), obesity, and allergies [1,2,3]. Among the factors that influence the gut microbiota, the most crucial and manageable is the animal’s diet. From raw diets and commercial kibble to protein-rich or fiber-enriched formulations, dietary interventions can reshape microbial communities and modulate host physiology [4,5].

This review provides an in-depth synthesis of the impacts of dietary components and formats on the gut microbiota in dogs and cats, and how these alterations are associated with health effects and the way we can support disease treatment through diet-based therapies. We also critically examine current limitations and propose avenues for future research.

**Figure 1:**
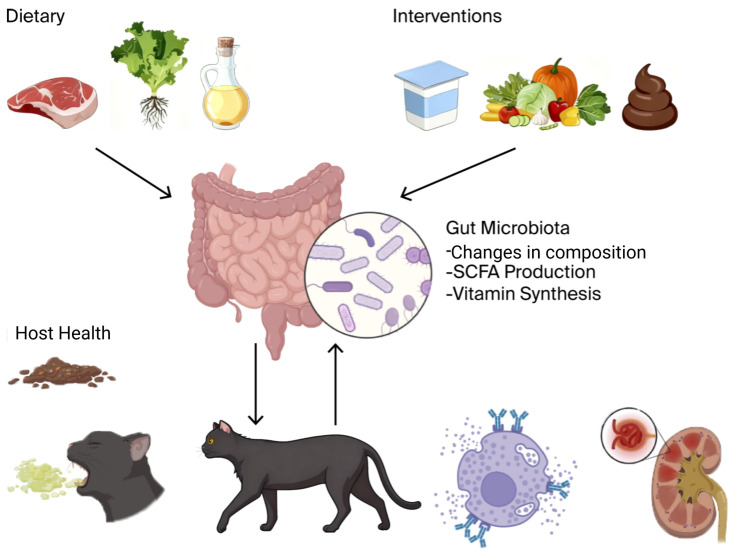
It is hypothesized that nutritional intervention has a direct effect on the activity and composition of the gut microbiota, alterations in the activity of which modulate a variety of host health outcomes, including metabolic and gastrointestinal illness as well as immune-linked and systemic diseases. The addition of adjunctive therapies (e.g., pre/probiotics, FMT) is reflected in the model, and the presence of feedback loops makes such a relationship dynamic. This figure was created with BioRender.com (version 2025, Toronto, ON, Canada) (Unique figure URL: https://BioRender.com/lr02qxr, accessed on 6 November 2025).

To conceptualize these complicated interactions, Figure 1 illustrates the overarching paradigm of the “Diet–Gut Microbiota–Host Health” axis in companion animals.

## 2. Composition and Function of the Gut Microbiota in Companion Animals

### 2.1. Composition of the Gut Microbiome in Dogs and Cats

The gut microbiomes of dogs and cats are dominated by a core group of bacterial phyla, including Firmicutes, Bacteroidetes, Fusobacteria, and Proteobacteria [6]. These microbial communities are largely shaped by diet, age, and other anthropogenic factors such as living conditions and human–animal interactions [4,7]. While the general structure of the canine and feline microbiome shares similarities with that in humans, species-specific microbial lineages suggest adaptations to the unique dietary and physiological needs of these animals [8].

### 2.2. Metabolic Functions of the Gut Microbiome

The gut microbiome has one of the most critical roles in metabolism: the decomposition of complex carbohydrates and non-digestible fiber that is not absorbed by the host [9]. These carbohydrates and fiber are digested by the gut microbes to yield short-chain fatty acids (SCFAs)—including acetate, butyrate, and propionate—which play indispensable roles in maintaining gut health and supporting metabolic functions [10]. SCFAs are not just an energy source to the host, but also regulate immune responses as well as the integrity of the intestinal barrier [11,12]. In addition, the gut microbiota helps in the biosynthesis of vitamins—including vitamin K and some B-vitamins—further indicating the metabolic role played by the gut microbiome.

### 2.3. Immune Regulatory Functions

The gut microbiome also plays a central role in immune regulation. Some bacterial groups—e.g., those of the genus *Prevotella* and family Ruminococcaceae—have been found to enhance immune function by elevating the production of anti-inflammatory cytokines and regulatory T cells [13,14]. An imbalance in the microbial communities, known as dysbiosis, can result in impaired immune function and the potential occurrence of chronic inflammatory diseases (e.g., IBD, autoimmune diseases) [15].

### 2.4. Digestive Functions

The gut microbiota is indispensable for digestion, particularly in breaking down complex dietary fibers and other indigestible carbohydrates [16]. The fermentation of fiber by gut microbes produces SCFAs, which are crucial for gut motility, the absorption of minerals, and the prevention of intestinal pathogens [17]. Cats and dogs experiencing gut dysbiosis normally present with gut-related symptoms such as diarrhea, vomiting, and pain in the abdomen [18,19]. Studies have also demonstrated that having pets positively impacts the gut microbe health of the animal as well as the owner, thus being favorable to the gut’s ability to assimilate nutrients as well as digest food [20].

### 2.5. Barrier Protective Functions

Beyond immune control and nutrient absorption, the gut microbiome functions as a necessary protection mechanism in the form of a barrier. The microbial communities in the intestines suppress pathogen adhesion by competing with damaging microbes for nutrients and attachment sites in the intestinal lining [21]. SCFAs, which are produced when fibers are fermented, help to maintain the integrity of the intestinal barrier, playing a crucial role in preventing leaky gut syndrome and related issues such as systemic inflammation and autoimmune diseases [22,23]. Dysbiosis—which is normally mediated by antibiotics, malnutrition, or stress—can compromise the intestinal lining, increasing the risk of pathogenic infection.

## 3. The Impact of Diet on the Gut Microbiome

### 3.1. Nutritional Components and Their Impact on Gut Microbiota

#### 3.1.1. Protein

One of the most important elements of food that has the potential to affect the gut microbiome of both cats and dogs is protein, with its level and source (animal vs. plant) influencing both community structure and metabolism.

- High-Protein Diets: It has been shown that high-protein diets (and, in particular, those with a high animal protein content) can promote the growth of Bacteroidetes and Firmicutes [6,24]. These phyla are involved in protein metabolism and the breakdown of complex amino acids. Nonetheless, high-protein diets also favor the development of *Clostridium perfringens*, which is a potentially pathogenic bacterium, particularly in cats and dogs whose immunity is impaired [25].
- Protein Source: The origin of the protein also matters. Proteins derived from animal sources (e.g., fish, beef, and poultry) have been shown to stimulate the growth of useful microbes like *Lactobacillus*, which are beneficial for preservation of the gut barrier, as well as immune function. In contrast, plant-based proteins may support a different set of microbial taxa, including those associated with fiber fermentation and SCFA production [26,27].

#### 3.1.2. Fat

Fat in the diet affects the gut microbiota composition by modifying the microbial diversity and functionality, depending on the type of fat.

- High-Fat Diets: Cats and dogs that consume high-fat diets demonstrate elevated numbers of Firmicutes, which are involved in fat catabolism. Omega-6 and omega-3 polyunsaturated fats, typically found in fish oils, can regulate the gut microbiome by elevating the numbers of anti-inflammatory microbes such as *Lactobacillus* and *Bifidobacterium* [28]. Fats also appear to regulate the gut barrier by elevating the numbers of microbes that are producers of SCFAs, elevating the integrity of the intestines.
- Obesity and Dysbiosis: High volumes of a high-fat diet with high caloric content can induce dysbiosis—a dis-equilibrium among obesity-related microbes and good bacteria. In such cases, the numbers of *Bacteroides uniformis* and *Clostridium* species can rise, resulting in inflammatory events in the gut [29].

#### 3.1.3. Fiber

Another crucial factor in the development of the gut microbiome in dogs and cats is the fiber in the diet. Fiber feeds gut microbes, driving fermentation and the generation of beneficial metabolites.

Soluble Fiber: Soluble fiber, in the form of inulin and psyllium, is fermented in the gut by microbes to yield SCFAs; particularly butyrate, which plays a central role in gut health, serving as an energy source for colonocytes (colon lining epithelial cells) and the gut [30,31]. Numbers of the butyrate-producing bacterium *Faecalibacterium prausnitzii* tend to be increased by high-fiber diets, which enhances intestinal immunity and could lessen the threat of inflammatory diseases such as IBD [32]. In a crossover study of 18 healthy dogs fed 12 foods with 5–13% fiber, while only about a dozen species shifted, the metabolite changes associated with those species were much larger. The responses also varied strongly by dog, suggesting that fiber benefits are driven more by function than taxonomy and may need a personalized approach [33].

Insoluble Fiber: Insoluble fiber, which is present in vegetables, whole-grain food, and cellulose, has the potential to increase the richness and diversity of the microbiota by increasing the proliferation of Firmicutes and Bacteroidetes species—central actors in fiber digestion and SCFA production [34,35].

#### 3.1.4. Carbohydrates

The structure and activity of the microbiota are affected by the solubility and complexity of carbohydrates, as well as carbohydrates in general. Highly digested carbohydrates or simple sugars vary in their effects, when compared to resistant starch and complex carbohydrates.

High-Carbohydrate Diets: A high intake of simple sugars and high-glycemic carbohydrates often raises the abundance of Proteobacteria and *E. coli*, which are associated with intestinal inflammation. This can worsen ailments such as obesity and diabetes [36]. On the other hand, carbohydrates with a low glycemic index (e.g., sweet potatoes or oats) may be a better choice and might enable the maintenance of a less pathogenic microbial community balance [37].

Resistant Starch: This type of carbohydrate, which can be found in green bananas and legumes, is classified under the term “resistant starch” as it is not digested in the small intestine, instead being fermented by bacteria present in the colon. It encourages the formation of healthy bacteria such as *Bifidobacterium* and *Lactobacillus*, and potentially helps to enhance the well-being of the gut and immune system [38,39].

### 3.2. Food Formats and Their Effects on Gut Microbiota

The format of the food (e.g., raw, cook-at-home, or commercial kibble) also affects the composition of the microbiome in companion animals [40].

#### 3.2.1. Biologically Appropriate Raw Food (BARF) Diets

The raw vegetables, bones, and raw meat in the BARF diet (Biologically Appropriate Raw Food) contain the same nutrients as those found in raw meat. This diet is thought to replicate wild canid and felid diets, and has been associated with an increase in microbial diversity in the gut.

Microbial Diversity: Dogs and cats that are fed a raw food diet present higher microbial richness and diversity compared to their equivalents fed a commercial diet. Studies have shown that the dogs on a BARF diet possess higher numbers of the useful bacteria *Lactobacillus* and *Bifidobacterium*, which are responsible for gut health as well as immune and gut barrier protection [40,41].

Risks of Pathogen Exposure: Although the microbial diversity in dogs fed a raw diet is usually advantageous, the abundance of opportunistic pathogens—including *Clostridium perfringens*—may also be elevated [42]. This implies that raw-food diets may cause both beneficial and adverse effects to the microbial balance, depending on lifestyle factors (e.g., hygiene) and dietary diversity [43,44]. Consistent with a more meat-based niche, feral cats showed higher microbial functional capacity than domestic cats, especially for amino acid and lipid degradation. A behavior-anchored model also linked the microbial production of short-chain fatty acids, neurotransmitters, and vitamins with higher aggression, hinting at diet–microbe–behavior coupling [45].

#### 3.2.2. Home-Cooked Meals

Home-cooked food can be prepared using more controlled ingredients; thus, such a diet is a widely used alternative to commercial diets among pet owners. Home-cooked foods are usually made with fresh, whole ingredients, including meats, vegetables, and grains, offering a highly digestible source of nutrition.

Microbial Composition: Home-cooked diets may increase the abundance of beneficial taxa such as *Bifidobacterium* and *Lactobacillus* [46]. Nevertheless, the balance of Firmicutes and Bacteroidetes under a home-cooked diet can significantly decrease according to the dietary composition of the home-cooked meals [5,47].

Consistency in Nutrients: It can be challenging to ensure that a home-cooked diet is nutritionally balanced. A deficiency in fiber or unsaturated fats may contribute to a lack of balance in microbe populations, leading to a less diverse gut microbiome [48].

#### 3.2.3. Extruded Commercial Kibble

The most common type of pet food is commercial kibble diets, which are normally processed by extrusion (a heat process used to shape the food). These diets are made out of dry and stable foods, and are normally enriched with vitamins and minerals. The dominance of kibble diets reflects their practical advantages: they are easy to portion and store and can reliably meet baseline nutrient targets at scale, while heat processing lengthens their shelf life while lowering the risk of household pathogen contamination compared with raw diets. Such diets are also the main carrier for “therapeutic” formulas, in which the energy density, protein-to-calorie ratio, fiber architecture, and add-ons can be tuned for the purposes of weight control, renal support, skin care, and more [49].

Microbial Shifts: Comparisons between raw and commercialized extruded diets showed that dogs fed extruded kibble often present lower alpha-diversity and a higher prevalence of Proteobacteria and Firmicutes [41]. Certain kibbles have high fat and carbohydrate contents, which can stimulate the development of pathogenic microbes that can affect the immune system, thus predisposing dogs to inflammatory diseases such as IBD [44]. In dogs, one month on a Western-style diet (i.e., high in fats and refined carbohydrates and low in fiber) increased colonic NF-κB activity, epithelial apoptosis, serum hs-CRP and MPO levels, and mucosal bacterial load, with a marked rise in fecal cholic acid. These inflammatory and bile acid shifts occurred without weight gain, indicating a direct dietary effect [50]. Multi-omics comparisons have likewise revealed differences between long-term kibble and raw cohorts in terms of the gut microbiome and serum metabolome; as kibble-fed groups often carry higher body condition scores, rigorous analyses were adjusted for BCS before attributing any effects to diet, and a balanced formulation (not simply “grain-free” vs. “with grain”) remains a key modifier [51,52,53].

Digestibility and Absorption: The extrusion process may reduce the bioavailability of some of the nutrients, which may in turn influence the growth of microbes. However, expensive kibbles containing added supplemental prebiotics and probiotics can maintain the natural balance of the microbes and minimize the effects of processing [54]. Head-to-head studies have shown that extruded kibble often has lower apparent total-tract digestibility (dry matter, protein, fat, energy) and higher fecal output/energy than fresh or low-processed diets, i.e., less usable nutrition per bite. High-temperature processing is linked to greater exposure to advanced glycation end-products (AGEs), whereas some low-processed diets yield lower serum AGEs; therefore, whether a given kibble mitigates AGE exposure depends on its process controls and antioxidant system. Practically, a diet should be chosen based on its energy density, protein quality, density (g protein per 1000 kcal), measured digestibility (ATTD/ME), fiber type, and manufacturer-disclosed processing metrics (e.g., AGEs, oxidative stress markers), rather than price or marketing terms [51].

## 4. Links Between Gut Microbiota and Disease in Dogs and Cats

### 4.1. Gut Microbiota and Gastrointestinal Diseases

The gut microbiota is crucial in canine and feline gut health. It helps in the digestion of food, maintenance of metabolism, production of essential nutrients, and protection against pathogens. Dysbiosis—namely, the imbalance or functionally aberrant structure of the microbiota—has been implicated in various gastrointestinal diseases in both species, including IBD, gut inflammation, and enteropathies of a chronic nature [3].

#### 4.1.1. Gut Microbiota and IBD

IBD is a widespread condition in dogs and cats, which is marked by chronic inflammation of the intestines. Research has demonstrated that gut dysbiosis is a major characteristic of IBD in companion animals. In IBD-affected animals, the abundance of beneficial bacteria (Firmicutes and Bacteroidetes) has been shown to decline while that of potentially harmful Proteobacteria increased [55,56]. These microbial changes are the cause of the intestinal inflammation characteristic of IBD [57]. IBD has also been associated with narrowed diversity in *Clostridium* clusters, suggesting that these bacteria play a key role in gut health.

#### 4.1.2. Gut Microbiota and Gastrointestinal Inflammation

The gut microbiome of dogs and cats with acute and long-term inflammation of the gastrointestinal tract has been found to vary significantly in relation to healthy animals [3,58]. In both species, inflammation can result in a shift in the microbial community, such that the share of pathogenic species rises and the share of protective species diminishes [8,59]. It has been identified that the alterations in the microbial community may aggravate inflammation and disrupt gut functionality, resulting in additional gastrointestinal problems [18].

### 4.2. Gut Microbiota and Chronic Diseases

It is being established that the gut microbiota plays a role in long-term illnesses such as obesity, diabetes, and heart diseases. Such states occur frequently in dogs and cats, and the correlation between the dysbiosis and these conditions is a developing field of study.

#### 4.2.1. Gut Microbiota and Obesity

Obesity is a frequent illness in dogs and cats, which typically leads to other metabolic illnesses. The gut microbiota has been related to the control of obesity due to their role in the process of digestion and metabolism, and the microbiota of metabolically unhealthy obese (MUO) cats has been found to differ from that of metabolically healthy obese (MHO) cats [60,61]. In particular, the number of *Ruminococcaceae* was higher in MUO cats, which was correlated with the presence of triglycerides and cholesterol, while other bacterial families such as *Bifidobacteriaceae* were observed to be correlated with healthier metabolism.

It has also been evidenced that the microbial composition in dogs with obesity is modulated in response to nutritional therapies, causing an increase in microbial diversity and metabolic health control. The shift in nutritional profile—in particular, in the protein–carbohydrate ratio—can directly influence the microbial communities and the treatment of obesity in companion animals [5,62,63].

#### 4.2.2. Gut Microbiota and Diabetes

Another long-term disorder is diabetes—specifically, insulin resistance and type 2 diabetes—which is associated with the gut microbiota [64]. Probiotics and selected nutraceuticals may modulate dysbiosis and improve insulin sensitivity in some contexts [65]. The dysbiosis observed in diabetic animals is typically characterized by inflammation, insulin insensitivity, and metabolic dysfunction; thus, microbiome modulation presents as a possible therapeutic tool for this condition.

### 4.3. Gut Microbiota and Immune-Related Diseases

The gut microbiota has been found to play a crucial role in the immune system by maintaining immune tolerance and regulating immune responses. Most immune diseases, including allergies, autoimmune diseases, and inflammatory diseases, have been shown to potentially correlate with dysbiosis [66].

#### 4.3.1. Gut Microbiota and Allergies

Atopic dermatitis and food allergies, which are allergic diseases, are also frequently observed in dogs and cats. Gut dysbiosis has been suggested to be involved in the pathogenesis of these diseases. New information demonstrates that intestinal dysbiosis can influence immune homeostasis, leading to predetermination and enhancement of allergic responses in animals. It is noteworthy that probiotics and prebiotics are nutritional interventions which have been demonstrated to possibly establish microbial balance and enhance immunological tolerance, thus serving as a possible solution to decrease the occurrence of allergies [67]. Such mechanistic insights—i.e., the modulation of systemic immune responses and inflammation by gut microbes—indicate the therapeutic potential of microbiota-targeted interventions in the treatment of canine and feline allergies [68].

#### 4.3.2. Gut Microbiota and Autoimmune Diseases

The gut microbiome and its connection with the autoimmune diseases IBD and osteoarthritis have garnered increasing attention. Mechanistic studies have indicated that the intestinal microbial communities actively regulate systemic immune responses, including T-cell differentiation and cytokine production [69,70]. In addition, new research has suggested that intestinal dysregulation mediated by microbiomes is not limited to intestinal diseases, but also contributes to the etiology of extra-intestinal autoimmune diseases in canine and feline hosts (including asthma and myasthenia gravis) [65]. Fresh insights into the etiological complexity of autoimmune diseases in a wide variety of organ systems are actively being developed, based on this emerging knowledge of microbiome–immune crosstalk.

### 4.4. Gut Microbiota and Liver and Kidney Diseases

Liver and kidney diseases in dogs and cats are also related to gut microbiota disturbances. Recent studies have confirmed that the gut–kidney and gut–liver axes take part in the pathogenesis of certain diseases.

#### 4.4.1. Gut Microbiota and Liver Diseases

The liver is involved in the body’s metabolic functions and is part of the gut microbiota’s close relationship with the body. Gut dysbiosis has been responsible for inducing liver diseases such as hepatic lipidosis and liver failure. Research has indicated that microbial-derived metabolites may influence liver health and microbial imbalances can exacerbate liver damage [71]. Despite being in an early stage, studies on gut microbiota manipulation as a function of nutritional intervention and probiotics have demonstrated its promising potential for the treatment of canine and feline diseases of the liver. In dogs, one month on a Western-style diet (high in fat and refined carbohydrates and low in fiber) increased colonic NF-κB activity, epithelial apoptosis, serum hs-CRP and MPO, and mucosal bacterial load, with a marked rise in fecal cholic acid. These inflammatory and bile acid shifts occurred without weight gain, indicating a direct dietary effect [50].

#### 4.4.2. Gut Microbiota and Kidney Diseases

An increasing volume of evidence reveals the gut–kidney axis as a pivotal component in the pathogenesis of chronic kidney disease (CKD) in household companion animals, with intestinal dysbiosis serving as an important component in the pathogenesis of the illness. Microbial imbalances in the gut–kidney axis enhance the formation of uremic toxins and the breakdown of the metabolic homeostasis in the host, further impairing the function of the kidney [72,73]. Microbiome modulation using the identified mechanisms of targeted nutritional strategies, probiotic supplementation, and microbiota-oriented treatments is a promising treatment paradigm for managing CKD, which will potentially preserve renal function through mechanisms beyond conventional nutritional support [74].

## 5. Dietary Interventions and Gut Health Management

### 5.1. Probiotics and Prebiotics

Probiotics and prebiotics also play important functions in maintenance of the gut microbiota in cats and dogs, elevating their general well-being; specifically concerning GI functions, immune function, and gut barrier integrity [75,76].

Probiotics: Probiotics are live microbes that provide health advantages by enhancing microbial diversity and the development of desirable bacteria. It has been shown that certain probiotics (i.e., *Lactobacillus* and *Bifidobacterium*) can benefit the gut of dogs and cats by mitigating the disruption of microbial homeostasis due to the occurrence of dysbiosis. Probiotics help to reduce inflammation, improve digestion, and enhance immune responses by promoting the growth of beneficial bacteria that outcompete harmful microbes [77,78]. Moreover, probiotics have been identified to cause a decrease in intestinal symptoms such as diarrhea and IBD in the two species [79,80].

Prebiotics: Prebiotics are indigestible food constituents (typically fiber or oligosaccharides) that selectively enhance the activity and proliferation of beneficial gut microbes. Two exemplary prebiotics are fructo-oligosaccharides (FOSs) and inulin, which are added to diets in order to enhance the gut microbiota composition [81]. Prebiotics are foods weith good microbes, helping them to proliferate and produce more SCFAs (e.g., butyrate) that are essential for gut health [82]. Prebiotics have also been demonstrated to regulate microbial diversity in dog and cat studies, enhancing intestinal health by promoting beneficial bacteria and suppressing harmful bacteria [83]. Probiotics and prebiotics (collectively called symbiotics) also have synergistic effects, resulting in improved gut wellness and general well-being, as shown through long-term in vitro experiments.

### 5.2. Specialized Dietary Interventions

In the treatment of canine and feline obesity, a large body of evidence suggests that calorie-restricted formulations with high protein and fiber contents can be used to induce satiety and weight loss through lean body mass conservation [26]. It has also been shown that supplementation with L-carnitine raises the metabolic rate of lipids, whereas omega-3 polyunsaturated lipids efficiently reduce the inflammatory reactions that accompany obesity [84,85]. Effective weight management regimens typically involve rigorous portion management, regular eating plans, and complementary physical activity.

In cases of food hypersensitivity and chronic gastrointestinal diseases, there is emerging evidence that new protein sources (e.g., rabbit, duck, or kangaroo) or hydrolyzed protein diets can be used to avoid antigenic stimulation [86]; this approach is even more effective when leveraging the therapeutic efficacy of high-fiber formulations to promote gastrointestinal health, coupled with pro- and prebiotic supplementation to re-establish microbial balance. The average time needed for clinical implementation is 6–8 weeks of total nutritional control, with constant monitoring of symptoms to determine the effect of the treatment.

Nutritional therapies consisting of restricted protein, phosphorus, and sodium, plus omega-3 acids of marine origin, have been shown to delay the progression of renal function impairment in the treatment of progressive renal diseases [87,88]. The use of adjuvant therapy with B-complex vitamins, potassium, and antioxidant compounds—especially vitamin E—is useful in maintaining renal homeostasis and oxidative damage. Prescription renal diets that are highly palatable are the key to the successful implementation of such protocols to achieve long-term compliance.

Nutritional support in the context of hepatic disease involves the use of moderately restricted, highly digestible protein content with high proportions of carbohydrates and soluble fiber to bind enteric toxins. S-adenosylmethionine (SAMe) and vitamin E have been proposed as hepatoprotective agents, and frequent small-volume feeding regimens have been shown to reduce hepatic metabolic stress [89,90,91].

In the case of cardiac patients, the management of hypertension is based on sodium-restricted diets accompanied by supplementation of taurine and carnitine as myocardial supportive agents [92]. The anti-inflammatory activities of omega-3 acids have been shown to be highly beneficial for the heart tissues, and strict vigilance regarding the dietary composition can prevent the unnecessary intake of sodium—the main cause of hypertension in clinical practice [93,94,95].

High-fiber complex carbohydrate diets have also been shown to be effective in the management of diabetes, in terms of moderating postprandial glycemic responses and enhancing insulin sensitivity [96,97,98]. These nutritional strategies showed improved treatment effects when used in combination with appropriate insulin regimens, providing synergistic effects for metabolic management.

Formulations supplemented with omega-3 fatty acids and medium-chain triglycerides have been shown to provide benefits in the geriatric population, promoting the maintenance of neurological, musculoskeletal, and metabolic function in late life [99,100,101,102]. These holistic nutritional interventions have the potential to address the multivariate issues associated with aging in companion animals.

Finally, evidence-based nutritional therapy is one of the pillars of advanced veterinary practice, with the implementation of disease-oriented diets showing great promise to deliver the best clinical outcomes and quality of life. To ensure a successful implementation process, qualified veterinary nutritionists should be involved to achieve the best formulation, accurate dosing, and long-term follow-up. Future research directions are related to the sphere of personalized nutrition approaches, merging of multi-omics data, and elaboration of universal protocols for various pathological conditions in different species.

### 5.3. Fecal Microbiota Transplantation (FMT)

FMT is an effective therapeutic approach for the restoration of intestinal microbial homeostasis in companion species, which has special applicability in chronic gut diseases for which nutritional therapies fail [103,104]. An expanding range of research indicates that FMT may be employed to successfully treat core clinical features of chronic enteropathy such as diarrhea, vomiting, and abdominal pains while, at the same time, treating underlying intestinal inflammation by re-structuring microbial communities and correcting dysbiosis [105,106,107]. The clinical effect of FMT involves more than the alleviation of symptoms, as it promotes specific functional recovery regarding important beneficial species—including (but not limited to) *Peptoacetobacter hiranonis*, which plays an indispensable role in bile acid metabolism [108]. Considering how diet can rapidly shift these endpoints—for example, Western-style feeding increases bile acids and mucosal inflammation, whereas diverse fibers enrich SCFAs and polyphenol-derived metabolites—we recommend routine follow-up of bile acids and SCFAs as functional readouts alongside composition [50]. New data have also indicated that successful FMT generates a measurable boost in the abundances of beneficial genera, including *Clostridium* and *Collinsella*; furthermore, these microbial improvements are strongly associated with clinical outcome measures across species [109]. Notably, the microbial restoration that FMT triggers is coupled to functional metabolic enhancements, as in the case of secondary production of bile acids, which is frequently dysfunctional in patients with chronic enteropathy [108]. The research findings to date agree with the multifunctional role of FMT in the management of various gastrointestinal diseases, with the increases in useful taxa—such as *Fusobacterium*—helping in the immune regulation process as well as healing of the mucosa [110,111]. Both these studies place FMT as an integrative treatment that addresses both compositional and functional aspects of gut microbiome restoration in companion dogs and cats. We have presented an overview of the clinical studies related to FMT in cats and dogs in this paper, as summarized in Table 1.

## 6. Challenges and Research Gaps

Despite considerable progress in clinical research exploring the relationship between the gut microbiota and canine/feline nutrition, numerous deficiencies and concerns have been identified that are still in need of study.

First, most existing studies suffer from small sample sizes. The majority included fewer than 20 animals, which limits statistical power and may affect the reliability and generalizability of the research findings. This persistent “small-sample” problem stems from high per-subject costs (dedicated diet/husbandry, repeated follow-up sampling—often multi-omics—clinical assessments/imaging, owner compensation, logistics/biobanking), compounded by multicenter coordination, ethics/privacy compliance, and intensive veterinary staffing; furthermore, recruitment is difficult, adherence depends on home environments and owner cooperation, case accrual is slow, and attrition is relatively high.

Second, the study periods are inevitably too brief. Most intervention studies are less than 8 weeks in duration. These extremely short durations of observation are insufficient to assess long-term dynamics or stability.

The existing literature is typically methodologically based on 16S rRNA gene sequencing, and the integration of multi-omics data (e.g., metabolomics and transcriptomics) remains lacking. This over-reliance on 16S rRNA sequencing without integrated multi-omics restricts understanding of the activities and metabolites of microbes, making it difficult to establish the correlations between the microbial composition and the phenotypes of the host. Recent dog fiber interventions (paired metagenomics and untargeted metabolomics), feline CKD multi-matrix metabolomics, and feral cat function-first models outline a practical template to link structure, function, and phenotype. Future studies should perform standardized quantification of bile acids, SCFAs, and key neuroactive metabolites in order to track functional recovery [45,122].

The number of species is also noticeably unequal. Although cats and dogs differ considerably in aspects such as physiology, dietary needs, and microbial composition, dogs have been more extensively studied. This precludes the development of advanced nutritional approaches exclusive to cats, due to a lack of adequate scientific support.

Another outstanding issue is the lack of connection between microbiome data and health indicators. Other studies have merely detailed the changes in the microbial composition and did not simultaneously examine clinically meaningful variables (e.g., immune parameters and indicators of gut barrier functionality), thus rendering the biological connection between microbial shifts and health condition hard to determine.

Lastly, existing studies lack subject and environmental diversity. As experiments are typically based on laboratory cohorts of animals fed highly controlled diets under a similar genetic background and living conditions, serious problems in terms of generalizing the results to the highly heterogeneous real-world household pet population arise.

## 7. Future Directions and Opportunities

In future studies, the following points deserve consideration in order to advance clinical investigations regarding the gut microbiota and canine and feline diets.

Conduct long-term longitudinal studies and enlarge sample size: To achieve long-term follow-up and large sample sizes under constrained resources, a systematic, low-cost study framework can be implemented. Establish multicenter collaboration with harmonized eligibility criteria and designated laboratories to reduce between-site variability and share fixed costs; shift data collection to the home setting using mailed sampling kits and digital workflows to lessen travel burden and improve adherence; employ within-subject (pre–post) designs and shared control groups to detect effects with smaller cohorts; track a focused set of hard endpoints in phased fashion and strategically enroll higher-risk populations to accelerate signal detection; and, finally, preregister protocols and standardize data elements to enable natural pooling across sites and expansion of the effective sample size. Together, this integrated strategy makes large-sample, long-term research feasible at a manageable cost.

Enhance multi-omics data integration: Studies can go beyond analyzing single microbiomes by integrating metagenomic data along with multidimensional metabolomics, proteomics, and immune marker data to more effectively comprehend the interactions between microbial functions and host physiology.

Develop personalized nutrition strategies: Individualized nutrition plans for companion animals should be grounded in a rigorous scientific foundation: first, estimate the daily energy requirement (DER) from body weight and activity level, set protein intake according to life-stage needs, and select a complete and balanced diet compliant with AAFCO/FEDIAF standards [123,124]. Thereafter, achieve precise alignment between intake, physiologic demands, and clinical objectives by titrating four key modifiable levers—namely, protein density (g/1000 kcal), fiber profile (soluble vs. insoluble), moisture content, and EPA/DHA dose. Implementation may follow differentiated pathways, based on the animal’s health status and the owner’s capacity: complex cases are best managed with clinician-directed prescription strategies (e.g., hydrolyzed-protein diets from Royal Canin^®^ or Hill’s^®^ paired with a structured elimination protocol); meanwhile, when convenience is prioritized, individualized fresh-food subscription services (e.g., Nom Nom, Butternut Box) can be used alongside serial body-weight monitoring and adjustment of feeding amounts. Home-prepared diets should employ validated recipes together with a reputable balancing supplement under professional supervision; in this context, the most accessible option—a “base diet plus functional adjuncts” approach—adds defined components such as psyllium husk or fish oil to enable gradual, goal-directed modulation.

Address research gaps in feline studies: Consolidate microbiome research in cats, wholly addressing their physiological and metabolic peculiarities in order to close the most important knowledge gaps regarding nutritional interventions in this species.

Clinical-first, microbiome-informed evaluation: A clinical-first, microbiome-informed, tiered framework is recommended to rigorously evaluate dietary interventions in dogs and cats. Subjective observations should be converted into objective, quantitative endpoints: at the clinical level, track longitudinal body weight, body condition score (BCS), and muscle condition score (MCS), complemented by activity data; for gastrointestinal health, record stool consistency scores and defecation frequency; for dermatologic disease, use pruritus scoring (PVAS) and lesion scoring (CADESI-4); and, for renal/metabolic status, monitor symmetric dimethylarginine (SDMA), creatinine, serum phosphorus, and related chemistries. In practice, it is acceptable to focus initially on 2–3 core endpoints (e.g., weight + stool score + activity) with scheduled reassessment. Beyond single-taxon analyses, emphasis should be placed on functional marker panels. The canine/feline Dysbiosis Index—a qPCR-based composite quantifying seven key taxa—can be used to estimate ecosystem balance, with values >0 indicating dysbiosis. Additional indicators include the fecal secondary-to-primary bile acid ratio, short-chain fatty acid profiles, and fecal inflammatory proteins (e.g., calprotectin), while serum vitamin B12 and folate inform nutrient absorption and microbial function. Although no universal “gold-standard” panel exists, a modular evaluation can be assembled: begin with baseline follow-up (weight, body condition, symptom scores), add blood chemistry and fecal inflammatory markers as needed and, when indicated, incorporate targeted analyses such as the Dysbiosis Index and metagenomics. Objective efficacy criteria include weekly weight loss of 1–2%, stool scores stabilized within the target range, clinically meaningful reductions in pruritus scores, and decreased reliance on rescue medications.

## 8. Conclusions

Nutrition acts as a modulator and mediator of gut microbial health, and is a cornerstone in the prevention and treatment of diseases in cats and dogs. In diseases of the gastrointestinal tract, metabolism, kidney, liver, and immune system, individualized nutrition has repeatedly been shown to balance microbes and promote favorable clinical responses. High-fiber, prebiotic-rich diets enhance short-chain fatty acid production and mucosal integrity, while omega-3 polyunsaturated fatty acids exert anti-inflammatory and metabolic regulatory effects. Hydrolyzed or new protein diets decrease the volume of allergic food reactions, while protein- and phosphorus-restricted diets delay the developmental process of chronic kidney disease. Similarly, antioxidant-enhanced (e.g., vitamin E, S-adenosylmethionine) hepatoprotective diets aid in detoxification and tissue repair. The overall results support the view that dietary modulation is not only a form of supportive care, but also a primary treatment approach for optimizing gut–organ axis activity.

Nevertheless, to maximize the effectiveness of diet–microbiota interventions, several limitations must be overcome. The existing studies are generally limited and short-term, and the relevant literature presents an uneven distribution of research, with larger gaps in research focused on felines. Furthermore, numerous studies have focused on compositional changes in the microbiota without providing any association with metabolic or immunological results. It is essential that subsequent research is longitudinal and multi-omics in scope, incorporating metagenomics, metabolomics, and immunophenotyping in an attempt to delineate functional mechanisms. Personalized nutrition strategies tailored to individual microbiome profiles and health phenotypes represent the next frontier in companion animal health. Ultimately, the development of microbiome-informed nutritional guidance would revolutionize companion pet health through therapeutic veterinary practice, in that diet becomes an evidence-based and routine means of ensuring and maintaining the long-term health of dogs and cats.

## Figures and Tables

**Table 1:** FMT parameters and outcomes in dogs and cats.

Species	Indication	Delivery Route	Dose and Frequency	Donor Screening (Pathogens/AMR)	Preparation (Fresh/Frozen; Vehicle; Anaerobiosis)	Peri-Procedural Regimen (Pre-Treatment and Co-Therapy)	Follow-Up (Weeks)	Engraftment/Biomarkers	Adverse Events	Clinical Outcomes and Durability
Cat [109]	Chronic digestive signs	Oral capsule	50 capsules over 2 weeks	Not detailed	Lyophilized, oral capsule; frozen	None reported	2 weeks	ASVs shared with donor; taxa shifts	None reported	Microbiota shifted toward healthy; varied by signs/diet
Dog [112]	Atopic dermatitis	Oral	Single dose	Yes (not detailed)	Not specified	None	8 weeks	Shared ASVs correlated with clinical improvement	None reported	Improved CADESI and PVAS scores
Dog [113]	Healthy (safety study)	Rectal enema	Single 5 g/kg dose	Yes	Fresh, enema	None	4 weeks	No change in dysbiosis index or immune markers	Mild (vomiting, diarrhea)	Safe, no immunologic impact
Dog [105]	IBD	Rectal enema	Repeated over months	Yes	Fresh, enema	Steroids previously	Long-term	Microbiota resembled donor	None	Symptoms improved; no adverse events
Dog [110]	IBD	Rectal enema	Single (unclear frequency)	Not specified	Fresh, enema	Not specified	Weeks	*Fusobacterium* increased	None	Clinical signs improved
Dog [114]	Tylosin-responsive enteropathy	Oral capsule	Daily ×4 weeks	Yes	Oral capsules; frozen	Tylosin restarted prior	8 weeks	30% donor strain engraftment	None reported	Higher response vs. placebo, not significant
Dog [115]	Parvoviral diarrhea	Rectal enema	Daily ×8 days	Not detailed	Not specified	Symptomatic therapy	8 days + 2 month	Improved transplant retention	1 fever, 1 epistaxis	Faster resolution, fewer relapses
Dog [116]	Diabetes mellitus	Oral capsule	Daily (duration unclear)	Yes	Lyophilized oral capsules	Insulin	60 days	SCFA, bile acids, Faecalibacterium ↑	None	Decreased water intake; mild benefit
Dog [117]	IBD	Rectal enema	Single	Yes	Frozen enema	Steroid + diet	30 days	No microbiota shift; safe	None	CCECAI↓ in both FMT and placebo
Dog [118]	AHDS	Rectal enema	Single	Yes	Not detailed	None	42 days	More stable DI than antibiotics	None	FMT not faster but more microbiota-stable
Dog [119]	Chronic diarrhea	Oral capsule	Multiple over time	Not detailed	Frozen capsules	Steroids	18 months	Symptoms improved; steroid-free	None	Long-term control, no relapses
Dog [120]	Chronic enteropathy	Rectal enema	Single	Yes	Not specified	None	12 months	CIBDAI ↓; propionate & bile acid metabolism ↑	None	Durable improvement in 50%
Dog [121]	Chronic GI signs	Oral capsule	50 capsules	Yes	Lyophilized oral capsules	Not specified	2 weeks	18% donor ASV engraftment	None	SCFA producers ↑; donor overlap affected outcome

Abbreviation: ASV—Amplicon Sequence Variant; AMR—Antimicrobial Resistance; DI—Dysbiosis Index; CIBDAI—Canine Inflammatory Bowel Disease Activity Index; CADESI—Canine Atopic Dermatitis Extent and Severity Index; PVAS—Pruritus Visual Analog Scale. Footnotes: Engraftment: Detection of donor-derived microorganisms or genetic signatures in the recipient’s stool after FMT, persisting beyond the immediate post-procedure sample and distinct from baseline. Durability: The length of time a predefined state is maintained without rescue therapy. ↑: Increase; ↓: Decrease.

## Data Availability

No new data were created or analyzed in this study. Data sharing is not applicable to this article.
